# COVID-19 Crisis: How Can Plant Biotechnology Help?

**DOI:** 10.3390/plants10020352

**Published:** 2021-02-12

**Authors:** Md. Jahidul Islam Shohag, Farhana Zerin Khan, Lin Tang, Yanyan Wei, Zhenli He, Xiaoe Yang

**Affiliations:** 1 College of Environmental and Resources Science, Zhejiang University, Ministry of Education (MOE) Key Laboratory of Environmental Remediation and Ecosystem Health, Hangzhou 310058, China; islam@zju.edu.cn (F.Z.K.); lin.tang@slu.se (L.T.); 2 Department of Agriculture, Bangabandhu Sheikh Mujibur Rahman Science and Technology University, Gopalganj 8100, Bangladesh; 3 Indian River Research and Education Center, Institute of Food and Agricultural Sciences, University of Florida, Fort Pierce, FL 34945, USA; 4 Cultivation Base of Guangxi Key Laboratory for Agro-Environment and Agro-Products Safety, College of Agriculture, Guangxi University, Nanning 530004, China

**Keywords:** biopharmaceuticals, COVID-19, diagnostics and therapeutics, plant biotechnology, transient expression

## Abstract

The emergence of the COVID-19 pandemic has led to significant public health crisis all over the world. The rapid spreading nature and high mortality rate of COVID-19 places a huge pressure on scientists to develop effective diagnostics and therapeutics to control the pandemic. Some scientists working on plant biotechnology together with commercial enterprises for the emergency manufacturing of diagnostics and therapeutics have aimed to fulfill the rapid demand for SARS-CoV-2 protein antigen and antibody through a rapid, scalable technology known as transient/stable expression in plants. Plant biotechnology using transient/stable expression offers a rapid solution to address this crisis through the production of low-cost diagnostics, antiviral drugs, immunotherapy, and vaccines. Transient/stable expression technology for manufacturing plant-based biopharmaceuticals is already established at commercial scale. Here, current opinions regarding how plant biotechnology can help fight against COVID-19 through the production of low-cost diagnostics and therapeutics are discussed.

## 1. Introduction 

**Figure 1:**
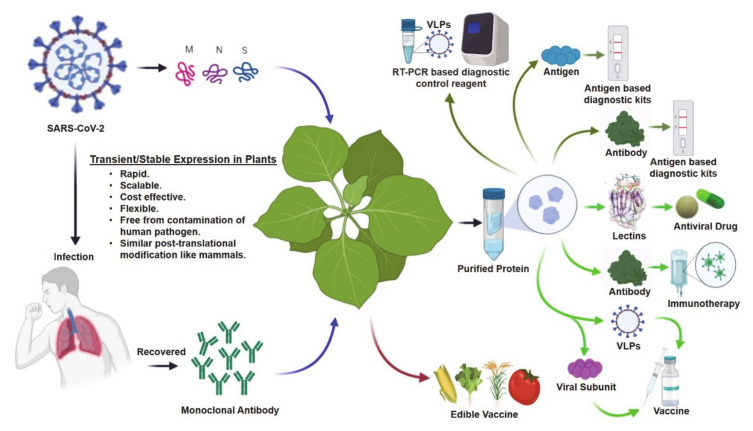
Developmental routes for plant-based diagnostics and therapeutics to address the COVID-19 crisis. Blue arrows indicate transient expression; brown arrow indicates stable expression; olive green arrows indicate potential routes for diagnostics; and light green arrows indicate potential routes for therapeutics manufacturing platforms. SARS-CoV-2: severe acute respiratory syndrome coronavirus 2; VLPs: virus-like particles. Some of these images were generated using Biorender (https://biorender.com/).

The COVID-19 pandemic is an urgent global health crisis in human history, which has dramatically affected the health, economy, and social mobility of almost everyone on the planet. Until now, more than 2 million people have died, and more than 93 million people have been infected with COVID-19 all over the world [1]. The normal life and livelihood of most world citizens have been disrupted, causing an incalculable economic depression. Health officials and government agencies have imposed extreme measures to limit human mobility and social distancing strategies to slow down the infection rate, thus decreasing the total number of hospitalized patients at one time. This strategy also allows more time to find and develop effective testing reagents to identify carriers, find suitable antiviral drugs to treat severely affected patients, and develop a vaccine to protect the unexposed portion of the population. Despite these measures, the COVID-19 pandemic has rapidly led to a serious crisis for diagnostic reagents and therapeutics, placing massive pressure on the supply and distribution network all over the world. During this crisis, plant scientists can play a key role in developing new diagnostics reagents and therapeutics with their knowledge and plant-based biopharmaceuticals infrastructure Figure 1).

Plants started being used as a biopharmaceuticals platform for the manufacturing of diagnostic reagents and therapeutic proteins immediately after the first successful expression of recombinant antibody in a plant [2]. Plants offer huge advantages compared with platforms based on traditional mammalian cell cultures. Using a transient expression system mediated by agroinfiltration and/or viral vectors [3,4], recombinant protein engineering and production in transgenic plants can be possible within 2 months after receiving the corresponding RNA sequence [5]. This speedy production system can rapidly address any pandemic crisis like COVID-19, as impressively shown by the production of an antibody cocktail for Ebola virus disease [6]. 

The cultivation of transgenic plants is relatively easy and cost effective (~0.0024 $ L^−1^), and only a specific nutrient solution and some basic infrastructure are required for growing transgenic plants [7], which is different from the high priced culture media required for mammalian cell cultures, which cost about 59 $ L^−1^ [8]. Under controlled environmental conditions such as greenhouse [9,10] combined with vertical farming systems [11,12], a multi-tone scale production of recombinant proteins may be possible within few months [13]. Furthermore, transgenic plant cultivation can be expanded into the agricultural field, which can easily produce recombinant proteins at a multi-tone scale. In addition, transient expression in plants is inherently safer because no human pathogens infect plant systems; therefore, plants are free from human pathogens, and there is no chance of contamination during gene transformation and production [14]. Additionally, plant systems are better than prokaryotic ones for complex protein expression such as monoclonal antibodies (mAbs) or membrane protein expression [15], because can perform post-translational modifications (e.g., glycosylation) similar to mammalian cells [16]. 

Using transient expression systems, plants may offer the only platform that can be used to produce diagnostics and therapeutics at a large scale in a few weeks, which is extremely relevant to the current pandemic situation, and they can be scaled up rapidly to address unforeseen and sudden demands. Several biopharmaceutical companies, for example, Protalix (Carmiel, Israel), Bayer (Halle, Germany), Planet Biotechnology (Hayward, CA, USA), Ventria (Fort Collins, CO, USA), Fraunhofer CMB (Newark, DE, USA), and Medicago (Quebec, QC, Canada), Agrenvec (Madrid, Spain), Diamante (Verona, Italy), ORF Genetics (Kópavogur, Iceland), Ventria Bioscience/Invitria (Fort Collins, CO, USA), and Mapp Biotherapeutics (San Diego, CA, USA), specialize in the production of plant-based proteins for diagnostics and therapeutics. Among them, some companies are in an advanced stage, conducting clinical trials to produce COVID-19-related diagnostics and therapeutics (Table 1), some of which will reach the market in the near future. Here we present current opinions in four areas where plant biotechnology could make huge contributions to address the current COVID-19 crisis: diagnostics to distinguish infected and recovered populations, antivirals to treat symptoms, antibodies to promote recovery of seriously affected hospitalized patients, and vaccines to protect un-infected populations.

## 2. Applications of Plant Biotechnology to Address COVID-19 Crisis

Since the hunter-gatherer age, humans have suffered from several infectious diseases. Plants have been historically recognized as the main source of therapeutics since that time. For centuries, aboriginal peoples all over the world have used traditional herbal medicine to treat infectious diseases. By contrast, the rise of the modern pharmaceutical industry in the past century has been based on exploiting individual active compounds with precise modes of action. In the last 20 years, plants have become vital competitors to bacteria-, yeast-, and mammalian-cell-based production systems for biopharmaceuticals. Plants are highly efficient in producing proteins of varying complexity, serving as a bioreactor/mini factory for manufacturing protein-based diagnostics and therapeutics. Plant-based biopharmaceutical production platforms exhibit agility, accuracy, and speed by eliminating the risk of mutation and contamination during production and significantly shortening production timelines. Plants have much to offer for fighting against COVID-19.

### 2.1. Diagnostics

Due to its rapid spreading nature, the COVID-19 pandemic has created a sudden huge crisis for diagnostics, and consequently caused severe shortage in the diagnostic reagents and materials to manufacture them. Currently, two types of diagnostics are in high demand. The first one is the antigen test to detect the virus directly and thus identify, separate, and treat the infected populations. The second one is the antibody test to detect the antibody produced against the viral infection and thus identify the infected, convalescent, and immune populations. There are two types of antigen tests: the first one based on the detection of viral genomic RNA, and the second based on the detection of viral proteins. In the RNA-based test, the virus is detected by quantitative reverse transcription PCR (RT-qPCR), for which we only need to synthesize gene-specific primers from the published genome sequence of SARS-CoV-2. However, a major problem with the RT-PCR-based test is the lack of a universal positive control; thus, there is a possibility of false positive or false negative results. This problem can be solved by developing plant-derived virus-like particles (VLPs) as a universal positive control for the RT-qPCR test. VLPs possess the same structure as the original virus but lack the native genome and are therefore unable to replicate. This technique was first applied to identify the foot and mouth disease virus [22]. Similar techniques can be applied to accurately detect the SARS-CoV-2-infected population using the RT-qPCR test. 

Identification of the corresponding antibody is the first step towards the development of diagnostics because the detection of viral protein requires specific ligands to bind. SARS-CoV-2 is made of four structural proteins; among them, the S protein could be a potential target for antibody-based detection because it plays a vital role in the virus entry into the host cell. Therefore, this protein could be used as a promising candidate for neutralizing antibody production. This can be achieved by injecting SARS-CoV-2 or the isolated or recombinant S protein/ receptor-binding domain (RBD) into mice to produce monoclonal antibody, or by screening phage antibody to display libraries using S protein/RBD. This procedure can yield the antibody sequence with high affinity for the S protein; as with this recombinant virus protein, transient antibody expression in plants can provide a rapidly scalable platform for production at commercial scale in a very short time, even with transgenic plants through a stable expression system, fulfilling the future need for longer-term commercial supply. The scaled-up production of such antibodies would allow the high-volume stockpiling of diagnostics for SARS-CoV-2 detection by using faster and more convenient formats, such as lateral flow assays (LFA), enzyme-linked immunosorbent assay (ELISA), or protein chips assay [23,24]. 

Sequence information of the SARS-CoV-2 can also be used to produce recombinant viral protein as a diagnostic reagent. The availability of SARS-CoV-2 recombinant proteins, especially the S protein, can be used for the development of diagnostics to detect serum antibody in infected and convalescent patients. Plants are the ideal means to produce these recombinant proteins within few weeks at a massive scale, so that diagnostics can be manufactured and stockpiled for supply and distribution with in a very short time. Therefore, transient expression systems can be used to address this crisis for rapid production and supply of diagnostic reagents, and further complemented by transgenic plants to achieve larger-scale production and longer-term supply of diagnostic reagents, as scientist are predicting that SARS-CoV-2 may persist as a seasonal flu for several years [25]. An Italian biotechnology company, Diamante, produced an ELISA kit for the detection of serum antibody in COVID-19 convalescent patients using tobacco plants to express SARS-CoV-2 RBD antigens [17].

### 2.2. Antiviral Drugs

Usually, viruses use similar metabolic pathways to those of the host cell to replicate and survive. These peculiarities of viruses hinder the development of appropriate antiviral drugs to suppress virus infection without any adverse effect on host cells. Antiviral drug development started after the end of the second world war with an increase of in vitro and in vivo studies on antiviral activity of medicinal plants [26]. Most antiviral drugs act against a specific viral enzyme that is involved in the replication cycle or the acquisition of the viral shape and therefore slow down the infection, and allow more time for the immune system to produce antibodies. Several plant extracts, isolated bioactive compounds, and essential oils, such as flavonoids, terpenes, coumarins, lignans, alkaloids, phenolic acids, and proteins, showed a vital role as antiviral drugs [27]. However, many conventional compounds have been found to be less effective against viral infection, and in some cases, the onset of specific viral resistance has led to an increasing interest for the plant-based production of protein as a promising antiviral drug [28]. 

Over the past decades, tremendous advances have been made in the standard use of proteins as antiviral drugs, especially carbohydrate-binding lectin protein from plants. Plant lectins are a heterologous class of carbohydrate-binding proteins, which are able to recognize and reversibly bind to the glycan structure present on the viral outer surface. A study on 33 plant lectins found that 20 of those candidates showed some activity against SARS-CoV [29]. Griffithsin is a plant lectin that acts as an entry inhibitor against several viruses including HIV [30], Zaire ebolavirus [31], SARS-CoV [29], and MERS-CoV [32], for which no vaccine is currently available. Moreover, plant griffithsin has low cytotoxicity in humans, offering an effective therapeutic window for SARS-CoV-2, as it has proven activity against the CoV family and some other viruses. It is not possible to predict whether plant griffithsin inactivates SARS-CoV-2, but it was found that the S protein of SARS-CoV and SARSCoV-2 are highly conserved, with some unique glycan positions [33,34]. 

Similarly, scytovirin is a lectin found in cyanobacterium *Scytonema varium* [35] that is also active against several viruses, including SARS-CoV [36]. Mannose-binding lectins exhibits the strongest activity against SARS-CoV, indicating that high-mannose glycans are a potential target. Moreover, lectins specific for galactose, glucose N-acetylgalactosamine, and N-acetylglucosamine also showed potential antiviral activity. Thus, plants are used as a bioreactor to produce a variety of antiviral lectins, including griffithsin [37,38], cyanovirin-N [39,40,41,42], and cyanovirin-N fusion proteins [43], as well as co-expression of griffithsin and cyanovirin-N in the seeds of transgenic rice line [44]. Once a lectin is found effective against SARS-CoV-2, a transient expression system could rapidly scale up its production and supply within a few weeks to address the antiviral drug crisis. Moreover, transgenic plants could then be developed through a stable expression system to generate a permanent resource for future larger-scale production.

### 2.3. Immunotherapy

Recent findings [45,46] suggested that plasma from convalescent patients can decrease the severity of COVID-19 symptoms and speed up recovery. This antibody-mediated virus neutralization can also happen through “passive immunity,” which involves providing antibodies against the spike protein as a treatment to patients. Passive immunotherapy can be performed by removing the plasma from someone who was infected with SARS-CoV-2 and generated an effective immune response against it. However, several potential complications can be associated with this procedure, including exposure of infected patients to additional viruses; therefore, a safer and better alternative is to use highly characterized and purified recombinant antibodies as immunotherapy. Application of recombinant antibodies against SARS-CoV-2 could help to reduce the infection and allow the body proper time to develop its own antibodies. Plants could be used in this case to produce antibodies as a form of passive immunotherapy. This procedure was first successfully demonstrated by Mapp Biopharmaceutical (San Diego, CA, USA) during the Zaire ebolavirus outbreak in West Africa. A cocktail of three neutralizing antibodies known as ZMapp [47] was developed by the LeafBio commercial arm of Mapp Biopharmaceutical, and was approved because of its life-saving ability and the lack of any other alternatives [6,48]. Up to 10 mg antibody is needed per infected patient, which means that growing transgenic plants at a massive scale would be a cost-effective solution for the manufacture of such product. Medicago Inc. (Québec, Canada) recently announced their intention to use their plant-based platform for the rapid production of antibodies against SARS-CoV-2 in collaboration with the Infectious Disease Research Centre of Laval University, Québec, Canada [21]. These SARS-CoV-2 antibodies could potentially be used to treat severe infected hospitalized patients.

By addressing scientific, technical, and regulatory demands of good manufacturing practice (GMP), transgenic plants were developed for the large-scale production of the HIV-neutralizing human monoclonal antibody 2G12 and 2F5, as demonstrated in transgenic tobacco (*Nicotiana tabacum*) [9], maize (*Zea mays*) [49,50], and rice (*Oryza sativa*) [42]. A similar approach could be used for neutralizing antibody production against SARS-CoV-2. Furthermore, transgenic plants offer a scalable and inexpensive bioreactor that can produce multiple components in a single plant simultaneously, which is also important because multiple components may be required in the future to avoid the rapid emergence of SARS-CoV-2 strains resistant to a single antibody. This approach can be exemplified by a recent research where a transgenic rice line was developed to produce HIV-neutralizing antibody 2G12 along with two antiviral lectins [44]. Simultaneous expression in the transgenic rice line allows the crude seed extract to be used directly as a preformulated cocktail, also avoiding the costs of multiple downstream processes. Moreover, transgenic plants also can be used to produce therapeutic antibodies, which can inhibit the cytokine storm that follows SARS-CoV-2 infection in many of the most severe and fatal cases. Two therapeutic antibodies repurposed for the treatment of COVID-19 are Sarilumab/Kevzara and tocilizumab/Actemra, currently undergoing clinical trials for COVID-19 [17].

### 2.4. Vaccine

Vaccines are the most economical and effective way to control and prevent any infectious disease. Therefore, the development of an appropriate vaccine against COVID-19 is urgent. Recent investigations have demonstrated that SARS-CoV-2 structural proteins can stimulate neutralizing antibodies and enhance the CD4^+^/CD8^+^ T cell response [51]. SARS-CoV-2 consists of four structural proteins. Among them, the N protein is highly conserved in the CoV family, whereas the M and E proteins induce a weak protective response, indicating that the N, M, and E proteins are unsuitable for targets vaccine candidates [52]. Therefore, the S protein is the main target for vaccine candidates. However, the S protein of SARS-CoV-2 is divided into the S1 and S2 subunits. S2 is membrane-spanning and highly conserved (99%) in the CoV family, whereas the S1 subunit shows only 70% individuality to other strains of human corona virus, and the differences are mainly in the RBD, which facilitates virus entry by binding to angiotensin-converting enzyme 2 (ACE2) on the host cell surface [53]. Blocking viral entry to the host cells is a promising strategy to control infection, and most of the vaccines for the SARS-CoV have targeted the S1 subunit for this reason [54]. Manufacturing subunit vaccines based on individual proteins, producing either virus subunit antigens or VLPs, is a safer and quicker alternative than vaccines developed through conventional approaches using inactivated or attenuated strains.

Many subunit vaccine candidates for pandemic or seasonal strains of influenza have already been developed by transient expression in the tobacco plant. Vaccine antigens were produced with a deconstructed vector based on the tobacco mosaic virus delivered by the agroinfiltration technique with *Agrobacterium tumefaciens.* This technology ensures uniformly high levels of target protein expression in *Nicotiana benthamiana* and can produce a maximum of 200 mg of protein per kg of tobacco leaves within 3 weeks just after receiving the corresponding sequences. From the CoV family, only one previous report demonstrated that the S1 subunit of swine-transmissible gastroenteritis coronavirus (TGEV) expressed in *Arabidopsis thaliana* transgenic lines produces recombinant antigen-elicited TGEV-specific antibodies in mice, indicating that immunogenic CoV antigens can be expressed and produced in plants [55]. The S1 protein of SARS-CoV-2 is heavily glycosylated [33,34], and the glycans are a complex mixture of high-mannose configurations, making it necessary to express the recombinant S1 and RBD with N-terminal signal peptides, ensuring that the proteins are secreted to the endomembrane system [56]. It remains unclear whether the structural differences between complex glycans in plants and humans result in remarkable differences for the potentiality of a plant-made SARS-CoV-2 vaccine, although the glycans structure is fully conserved among the eukaryotes. Considering the above particulars of SARS-CoV-2, BAT’s US bio-tech subsidiary, Kentucky BioProcessing (KBP), is developing a potential vaccine antigen for COVID-19. In a pre-clinical study, it produced an immune response in the body and, in particular, the production of antibodies. This vaccine antigen would then be inserted into *Nicotiana tabacum* plants for reproduction, and once the plants are harvested, the antigen can then be purified to produce a subunit vaccine with the mixture of appropriate adjuvants. BAT is hopeful to manufacture around 3 million doses per week using their tobacco-plant-based platform [19]. Similarly, iBio (Bryan, TX, USA), a plant-based biologics manufacturing company, announced the production of a subunit vaccine (“IBIO-201”) produced from antigens derived from the SARS-CoV-2 spike protein fused with their booster molecule LicKM [20].

Virus-like particles (VLPs) based on plant viruses represent an exciting prospect for vaccine development. VLPs mimic the original structure of virus, which allows them to be easily recognized by the immune system of the host. VLPs lack core genetic materials, which ensures an extra layer of safety, as they cannot replicate in humans, making them non-infectious, and can be manufactured in huge quantities by transient expression in plants [57]. Using the tobacco plant as the production host, a VLPs platform has been made by Canadian biopharmaceutical firm Medicago Inc. (Québec, Canada), and they achieved the significant milestone of manufacturing more than 10 million vaccine doses against H1N1 influenza in 1 month, as part of the DARPA Blue Angel program [58]. At the beginning of the COVID-19 outbreak, Medicago announced the use of a similar plant-based production platform to manufacture the COVID-19 vaccine antigen; recently, they announced the successful production of VLPs. The technology involves the use of plant leaves as bioreactors to generate the S-spike protein that self-assembles into VLPs for the COVID-19 VPL (CoVLP) vaccine candidate. Medicago’s recombinant CoVLP mimics the SARS-CoV-2 structure and enables identification by the immune system. Using a plant-based production platform to manufacture the COVID-19 vaccine antigen, Medicago expects to be able to manufacture up to 100 million doses by the end of 2021, with a further 1 billion doses planned to be delivered annually by the end of 2023, following the completion of a large-scale facility currently under construction in Quebec City [21]. Similarly, iBio (Bryan, TX, USA) developed IBIO-200, a SARS-CoV-2 VLPs vaccine using the tobacco plant as a bioreactor. They will use their proprietary Fast Pharming system combined with an automated hydroponics plant culture, vertical farming, and glycan engineering technologies to rapidly deliver about 500 million doses annually [20].

## 3. Concluding Remarks

Plant biotechnology through transient/stable expression in plants offers an outstanding platform to produce biopharmaceuticals to fight against COVID-19. Transient/stable expression in plants is a faster, cost effective, scalable, and flexible technology than traditional microbe-, insect-, or mammalian-cell-based platforms because there is no need to establish stable culture cell lines or costly culture media, neither is there any need for an extra set-up for the scaled-up production except for the cultivation of more plants. Moreover, the development of stable transformation is also necessary to produce edible vaccines, which could find their place in the post-pandemic phase. Crop plants can be grown in diverse environments; therefore, biopharmaceuticals could be produced using already established infrastructures for agricultural production and the same distribution networks that exist for the supply of food and cereal seeds, without the need for a cold supply chain. Plant biotechnology has the opportunity not only to fight against COVID-19 but also to create a perfect model that allows a rapid and intended response to any crises in the future. 

## Figures and Tables

**Table 1:** Plant-based biopharmaceuticals under production to address the COVID-19 crisis.

Products	Company/Organization	Transformation Methods	Plant Host/Expression Systems	Status	References
Diagnostics					
RT-PCR based Diagnostic control reagent	John Innes Centre (Norwich, UK)	Agro-infiltration	*Vignan unguiculota*	Production	[17]
SARS-CoV-2 Nucleocapsid (N) Protein (Antigen based Diagnostic reagent)	Leaf Expansion System (Norwich, UK)	Hypertrans^®^ system	*Nicotiana benthamiana*	Production	[18]
Vaccine					
COVID-19 Subunit Vaccine (KBP-201)	Kentucky BioProcessing, Inc. (KBP)	Agro-infiltration	* Nicotiana benthamiana *	Phase II	[19]
COVID-19 VPL Vaccine (IBIO-200)	iBio, Inc (New York, NY, US)	FastPharming™ system	* Arabidopsis thaliana *	Pre-clinical	[20]
COVID-19 Subunit Vaccine (IBIO-201)	iBio, Inc (New York, NY, US)	FastPharming™ system	* Arabidopsis thaliana *	Pre-clinical	[20]
COVID-19 VPL Vaccine (CoVLP)	Medicago Inc. (Québec, Canada)	VLPExpress™ system	*Nicotiana benthamiana*	Phase III	[21]
Immunotherapy					
COVID-19 Antibody 1	Medicago Inc. (Québec, Canada)	Proficia^®^ system	*Nicotiana benthamiana*	Research	[21]
COVID-19 Antibody 2	Medicago Inc. (Québec, Canada)	Proficia^®^ system	*Nicotiana benthamiana*	Research	[21]

## Data Availability

Not applicable.
